# Impact of COVID-19 on the Quality of Life of Households in Saudi Arabia

**DOI:** 10.3390/ijerph19031538

**Published:** 2022-01-29

**Authors:** Md. Mazharul Islam, Majed Alharthi

**Affiliations:** Department of Finance, College of Business, King Abdulaziz University, Jeddah 21589, Saudi Arabia; malislam@kau.edu.sa

**Keywords:** COVID-19, quality of life, socio-economic and demographic group, Saudi Arabia

## Abstract

The COVID-19 pandemic has affected every stratum of the population and all categories of households to varying degrees. The impact of the pandemic on the quality of life (QoL) of populations is complex and can vary by region, socio-economic status, and other demographic factors. The main purpose of this study was to empirically examine the effects of pandemic trauma on the QoL of households in Saudi Arabia. Primary data from 506 households in different regions were collected through online surveys and estimated using descriptive statistics, analysis of variance (ANOVA), statistical regression techniques, and ordered Probit analysis. It was found that the QoL of Saudi households dropped significantly due to the COVID-19 crisis. Demographically, there were significant differences in the impact of COVID-19 on QoL. Low-income households, large households, male-led households, urban households, households living in the central and western regions, households with head unemployment or low educational attainment, and households with elderly head reported greater QoL declines. The findings emphasize the significance of generating on-the-ground survey data to track the well-being of households during the crisis to accumulate the information required to construct evidence-based policy responses. This study makes a significant contribution to the literature on the impact of COVID-19 by providing additional evidence of the pandemic’s impact at the household level. The study paints a grim picture of the effects of COVID-19, as it was carried out at a time when the coronavirus was spreading, millions were dying or fighting it in healthcare centers, and lockdowns were imposed throughout the world.

## 1. Introduction

The COVID-19 outbreak has caused considerable business, economic, and psycho-social disruptions worldwide. Due to the absence of pharmaceutical interventions, non-pharmaceutical approaches are the only way to prevent this disease, which considerably disrupts daily physical lifestyles, social interactions, and economic operations. Living conditions have changed around the globe as a result of COVID-19. In the short term, it has negatively affected the world economy, which has led to many misfortunes and which may have grave consequences in the long run. The World Health Organization (WHO) [1] stated that the economic crisis caused by COVID-19 has seriously negatively affected disadvantaged and vulnerable people. It is well recognized that the COVID-19 crisis in October 2019 had numerous harsh and unexpected impacts on all sectors. However, society is suffering more broadly through the loss of wages, jobs, and opportunities, deteriorating the progress made regarding quality of life (QoL) over the past decades. The WHO also reported that many enterprises are at risk of failing. During lockdowns, people worldwide were unable to feed themselves and their families because they were unable to earn an income. Hence, their QoL deteriorated drastically [1].

A number of studies have been conducted to determine the short- and long-term outcomes of this world-reaching outbreak. To the best of our knowledge, the majority of these studies focused on the macro-economic impacts of COVID-19 based on aggregate data. For example, in 2020, an equivalent of 400 million full-time jobs were lost worldwide [2], employees’ income dropped by 10% [3], investment decreased by 19% [4], global GDP shrank by nearly 22 trillion dollars [5], remittance flows decreased [6], and poverty and hunger increased [6,7]. However, aggregate data may ignore significant segments of the population and be inadequate, compared to simple surveys, in tracking the well-being of the poor [8]. Therefore, it is essential to conduct micro-level studies through direct surveys of households for a systematic and in-depth look at how COVID-19 has disturbed people’s lives during the worldwide epidemic. The WHO [1] clearly stated that the world has to live with the coronavirus. Hence, it is imperative to investigate the effects of COVID-19 on QoL. Understanding the impact of COVID-19 on QoL and the emotional and demographic factors shaping it will help governments establish a concrete and effective, comprehensive social safety net to protect all segments of the population. Existing micro-level studies have uncovered the impact of COVID-19 on individual aspects of QoL and have largely reported negative outcomes such as depression and anxiety [9], feelings of fear, stress, and worry [10,11], and psychological distress [12]. These findings regarding individual aspects of QoL do not cover the impact on overall QoL. Thus, to protect all segments of the population from any sudden shocks such as COVID-19, it is necessary to conduct empirical studies to discover the outcomes of COVID-19.

The authors attempted to fill this gap by empirically investigating the effects of COVID-19 on households in Saudi Arabia. The purpose of this research was to investigate the severity of these effects across different regions in Saudi Arabia, identify those most affected in terms of demographics, and recommend policies and programs for a reliable, comprehensive social safety net directly and indirectly linked with sustainable economic development in Saudi Arabia.

### Justification for Choosing Saudi Arabia

Since the main goal of Saudi Arabia’s Vison 2030 is to achieve sustainable economic development, the effects of the pandemic on society, the economy in general, and QoL in particular are a pressing issue among people in Saudi Arabia. As with other economies around the globe, the performance of the Saudi economy in 2020 was adversely affected by COVID-19. The immediate socio-economic impacts can be seen in falling gross national product, balance of payments, rising unemployment, falling income, reduced savings, sharp increases in the cost of living, increased crime, and lower living standards for large sections of the country [13]. One of the most obvious effects of COVID-19 is the contraction of the economy. For example, in Saudi Arabia, GDP decreased by 1.0% in the first quarter of 2020 and by 7.0% in the second. Negative growth rates of 10.1% and 3.5% were recorded in private and government sectors, respectively, and the unemployment rate reached 15.4%. A slowdown in key sectors continued these trends for the remainder of the year, and GDP was expected to contract by 5.4% in 2020 [6]. Notably, the effects of the economic crisis caused by COVID-19 have been observed to varying degrees among people in every stratum of society. However, the impact of COVID-19 on the QoL of households is complex, and it has varied by locality and among socio-economic and demographic groups. In particular, the QoL of low-income households deteriorated considerably due to decreased income levels and disadvantages in education, resources, and skills. Their vulnerability to economic, financial, and other crises made them an important target group for assistance. While some studies have focused on the socio-economic impact of the pandemic in Saudi Arabia, none have focused on affected groups. Furthermore, micro-level studies of the impact of COVID-19 are lacking. To establish a comprehensive social safety net, which is essential for sustainable development, micro-studies that analyze how different segments of society have been affected by COVID-19 and how they responded to aspects of the economic crisis should be conducted. Therefore, investigating the consequences of COVID-19 for the QoL of Saudi households is crucial for the provision of mitigation policies, strategies, and programs. Therefore, the goal of this study was to address the current void in the literature by researching the effects of the pandemic on household QoL in Saudi Arabia from a family perspective. To achieve this, we investigated the socio-economic conditions of households in Saudi Arabia suffering from the economic crisis caused by COVID-19. We also identified the factors that transformed the QoL of households. Finally, we recommend policies and programs for an effective safety net directly and indirectly linked with the goals of Saudi Arabia’s Vision 2030.

## 2. Literature Review

Regarding economic impacts, Scott [14] reported that the coronavirus outbreak has already had a much larger economic impact than either of its predecessors (MERS and SARS). For example, Egger et al. [15] conducted a study on nine low- and middle-income countries to examine how the COVID-19 outbreak affected the living standards of households and reported that households experienced high employment declination ranging from 5% to 49% (median, 30%), an income drop ranging from 8% to 87% (median, 70%), reduced access to the market (from 3% to 77%, median 31%), and an increase in food insecurity ranging from 9% to 87% (median, 59%). They also concluded that many households (median 48%) were unable to meet basic nutritional needs and therefore adopted coping mechanisms such as reduced sizes and numbers of daily meals. Consequently, their well-being and living standards dropped drastically across the socio-economic spectrum. The decline in well-being and living standards varied substantially by country and region due to cultural characteristics [15]. To reduce economic fallout, most countries have developed economic stimulus packages to mitigate the economic crisis caused by COVID-19. As with other countries, Saudi Arabia has announced an almost USD 61 billion stimulus package to minimize the impact [16].

COVID-19 has seriously affected both the pilgrimage and petroleum industries, on which the Saudi economy most heavily depends upon. COVID-19 also caused a complete shutdown of the country, including the closure of local and small businesses, restaurants, open food markets, service industries, food processing industries, and raw food supplies, which has affected the socio-economically disadvantaged. The price of food and other items has increased, many jobs have been lost, and huge cost reductions have been inevitable, which has affected development funds and humanitarian projects that ultimately impacted household incomes. These aspects of the pandemic have caused mental stress among different socio-economic groups, and low-income households are the most vulnerable. These unprecedented damages in human lives were unthinkable even a month before October 2019. In addition, prayer in mosques is almost a compulsory routine in the KSA and, therefore, detachment from mosques has had a psychological effect on Saudis.

Many studies have detailed the severity of COVID-19 and shown how individuals and the private and public sectors have faced the disease and its effects on society and the economy. For example, Gokmen et al. [17] discovered a relationship between the effects of COVID-19 and cultural characteristics. The results of their study indicated that egalitarianism, individualism, and indulgence had significant positive effects on the growth rate of COVID-19 cases (compared to the counterparts). They also found that egalitarianism was more important than individualism in predicting COVID mortality. Their findings were strongly supported by those of He et al. [18], Jiang et al. [19], Gelfand et al. [20], and Biddlestone et al. [21]. In contrast, Wang [22] reported that national culture did not have significant impacts on social distancing, which is considered a main factor in the growth rate of COVID-19 cases. Albulescu [23] tested the correlation between coronavirus cases and crude oil prices during the period from 21 January to 13 March 2020, using daily basis data. The results confirmed that the relationship between COVID-19 and oil prices was significantly negative and led to economic crises in oil-exporting countries. Moreover, McKibbin and Fernando [24] claimed that the coronavirus epidemic has severely damaged the world economy in the short term. Anderson et al. [25] claimed that world governments have been stunned by deaths and massive economic disruption due to COVID-19. Haushofer and Metcalf [26] suggested mitigating the effects of COVID-19 through behavioral economics. Qiu et al. [27] reported that the coronavirus pandemic has caused mental problems such as fear and misery in China. They also suggested that extra care should be provided to vulnerable groups such as low-income households and female workers. Lipsitch et al. [28] stated that urgent study is necessary to focus on numerous key issues: What is the full scale of COVID-19 severity within and across nations? Which demographics are most affected? Finally, Lazzerini and Putoto [29] argued that more empirical studies are needed to improve decision-making and encourage awareness.

However, few studies have been conducted at micro-levels to identify the effects of the pandemic on QoL. For example, Bukari et al. [30] conducted a study to uncover the consequences of the pandemic for poverty levels and the living standards of Ghanaian households. The results revealed that living standards have declined considerably with increased household poverty levels due to COVID-19. The study also showed that female-headed, rural, and unemployed households have been most affected. These findings are supported by van Dorn et al.’s [31] study conducted in the USA. According to them, the severity of the impact was greater on uninsured and underinsured people who lived in rural areas. This assertion has been strongly supported by other studies [32,33]. These studies suggest that an unbiased distribution of insufficient resources is essential, with underprivileged areas such as countryside populations covered, to overcome the impact of COVID-19.

Rabacal et al. [34] conducted a study in the Philippines to determine the impact of COVID-19 on teachers. Their study found a moderate impact of the pandemic on teachers’ QoL. They reported that the deterioration of QoL did not significantly differ by age, sex, marital status, employment status, monthly salary, presence of COVID-19 cases near their residence, personal knowledge of someone who was infected or died of COVID-19, presence of a medical condition, or perceived threat. Another study was conducted by Ping et al. [35] in China to evaluate the impact of COVID-19 on health-related QoL, using the EuroQol 5 Dimensions (EQ-5D). They concluded that QoL had significantly deteriorated among the elderly and people with lower income, low educational levels, chronic disease, and concern about COVID-19 infection. Concurrently, Epifanio et al. [36] conducted an empirical study to assess the impact of COVID-19 and lockdown measures on QoL in Italy. They reported a significant difference in the effects of COVID-19 on QoL by sex, area of residence, and diagnosis of a medical/psychiatric condition. They noted that those that were of a younger age, female gender, unemployed status, with a preexisting illness, and those living in the south of Italy were more affected by COVID-19 than others. The findings of Wenham et al. [37] supported this finding. Furthermore, the authors claimed that the effects of COVID-19 on women varied by employment status. However, Kharshiing et al. [38] reported that during COVID-19, QoL was not significantly impacted by demographic factors in India, but was significantly impacted by individual factors (notably, personal identity (such as personal standards, emotions and feelings, thoughts and ideas, and values and ethics) and COVID-19 anxiety) and social or group factors (identification with family, religious group, and nation).

Limcaoco et al. [39] and Wang et al. [9] stated that anxiety about COVID-19 significantly affected people’s QoL. Their findings are strongly supported by those of Kharshiing et al. [38], who reported that QoL was significantly impacted by individual factors such as anxiety, perceived susceptibility to and severity of COVID-19, optimistic bias, and personal identity. In contrast, Zhang and Ma [40] claimed that individual factors had no effect on the QoL of local Chinese residents. The changes precipitated by COVID-19 to people’s employment situation, work–life balance, and financial situation have greatly impacted their well-being [41].

**Figure 1:**
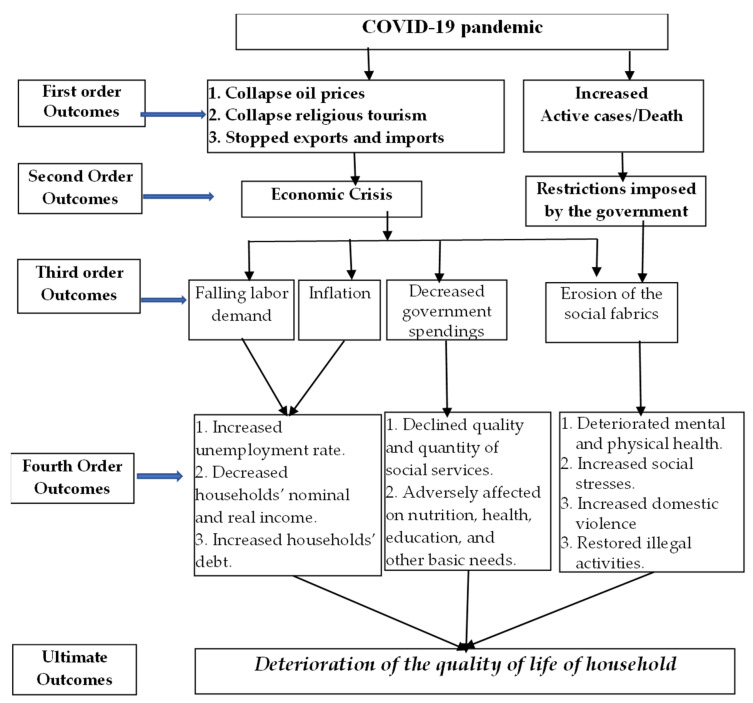
Transmission of the impact on QoL by COVID-19 at the micro-economic/household level.

Based on the literature, it is evident that the coronavirus pandemic crisis has been transmitted through various channels. A framework of the transmission flow of the impacts of the pandemic on QoL at the household level is shown in Figure 1.

Based on the existing literature, then, the following hypotheses were proposed for this study:

**Hypotheses** **1** **(H1).** *The economic crisis precipitated by COVID-19 had an adverse impact on the QoL of households in Saudi Arabia.*

**Hypotheses** **2** **(H2).** *The effects of COVID-19 on household QoL varied according to demographic factors such as age, gender, education level, residential area and region, income level, number of working family members, and family size.*

## 3. Materials and Methods

The systematic approach for empirical research suggested by Flynn [42] was used in this study. Research problems were formulated following an extensive literature review, and then a questionnaire survey was conducted to test the hypotheses.

### 3.1. Study Design

This quantitative study utilized a survey to test the hypotheses. A multivariate analysis was conducted alongside a descriptive snapshot. A regression analysis was then conducted to explore the demographic factors associated with changes in the QoL of Saudi households due to the COVID-19 crisis. For data analysis, the Statistical Package for Social Sciences (SPSS) version 25 was used. The specific regression model for QoL used took the following form:

Y_QoL_ = β_0_ + β_i_X_i_ + ε_i_

where Y_QoL_ stands for the household’s QoL variable (the average of six items on a 5-point scale, which was developed and validated by Repišti et al. [43]). A higher value of Y_QoL_ indicates that a household experienced greater deterioration of QoL since the COVID-19 pandemic started. X_i_ is a vector of explanatory variables, and ε_i_ refers to the error term.

In addition, ordered Probit models were run discretely to further investigate which demographic characteristics of respondents most significantly affect the aspects of QoL individually.

### 3.2. Questionnaire and Sample Design and Data Collection Procedure

Based on an in-depth literature review, survey instruments were developed, pre-tested, and validated by a focus group containing four individuals. The group comprised two academic experts from King Abdulaziz University, Jeddah, and two heads of households in Jeddah. Based on pre-testing, modifications were made to ensure the clarity of the questionnaire items. The format was confirmed as appropriate, as the survey was conducted in Arabic. The data were gathered electronically as an “online survey” from 1 January 2021 to 2 February 2021, from the heads of the households in various regions of Saudi Arabia. Social media, such as WhatsApp, Facebook, and LinkedIn, were used to circulate the survey and collect data. A consent letter was sent to the respondents to inform them of the purpose of the study and to assure them that their responses would be anonymous and confidential. A minimum of 10% of completed surveys were independently monitored and validated in real time by the project leader.

### 3.3. Measure of the Constructs

The QoL Program in Saudi Arabia is one of the most important programs required to realize Saudi Arabia’s Vision 2030 [44]. This program identifies that QoL can be achieved through promoting sports activities in the community, improving living conditions, diversifying, and developing entertainment and tourism sectors, enhancing the quality of services, and resistance to drug use [45]. The WHO has defined QoL as “individuals’ perceptions of their position in life concerning the culture and value systems in which they live and regarding their goals, expectations, standards, and concerns” [46]. Many studies have linked QoL with health, such as those by Bryson [47], Geirdal, et al. [48], Jones [49], and Rios, et al. [50], who have confirmed that QoL can be enhanced through focusing on human health. Studies have explained that QoL can be attained through social and economic positions [51,52], and they have concluded that higher social activities and personal economic growth has led to better QoL. Our study utilized six items that validated the construct of QoL (COV19-QoL), which was developed by [43]. All items were measured using a 5-point Likert scale ranging from 1 (completely disagree) to 5 (completely agree), and participants were asked to reflect on their feelings and thoughts for each item on the scale. The total score was calculated by averaging the scores for all the items. A higher score indicated a greater perceived impact of the pandemic on QoL. The content of the QoL scale was acceptable, and its construct validity was confirmed by convergent validity (factor loadings of respective measured items > 0.50) and discriminant validity (AVE > r^2^). Cronbach’s alpha was computed to be 0.744 (Table 1).

## 4. Results and Discussion

### 4.1. Profile of the Respondents

From the survey, 506 responses were received for an overall response rate of 50.6%. Ref. [53] categorized sample sizes in empirical studies as small (*n* < 100), medium (100 < *n* < 200), and large (*n* ≥ 200). Moreover, the sample-to-variable ratio model suggests a minimum observation-to-variable ratio of 5:1, but ratios of 15:1 or 20:1 are preferred [54]. This means that 15 to 20 respondents are strongly recommended to consider each independent variable in the model when determining the sample size for the research. This rule can also be used for multiple regressions and similar analyses. Accordingly, the acceptable sample must be 260 (20 × 13 (number of independent variables)). Therefore, the sample was considered large and suitable for analysis. In addition, the study employed a bootstrapping method (with *n* = 5000 bootstrap resample). In addition, Hair et al. [55] stated that a response rate of more than 50% is highly acceptable for survey-based research. Table 2, Table 3, Table 4 and Table 5 shows that approximately 71% (360) of the respondents were male-headed households and 29% (146) were female-headed households. Approximately 58% (294) of the households were middle-income earners, while 36% (180) and 6% (32) were low-income and high-income earners, respectively (the income classification used by Alnuaim [56] was applied. Accordingly, income lower than 3800 SR is referred to as low income; income from 3800 to 38,200 SR is referred to as middle income; and income higher than 38,200 SR is referred to as high income). For residential areas, the proportion of respondents was 56% (283) and 44% (223) in urban and rural areas, respectively. The ages of most of the heads of households (49%) were between 35 and 54 years, and 49% (247) of households had 5 to 10 family members living together. Regarding type of occupation, 33% (168) of the respondents were employees, 13% were unemployed (66), 29% were self-employed (149), 16% were employers (81), and 9% were retired (42). For education level, 38% (192) of the respondents had bachelor degrees, 22% (110) had secondary school-level degrees, 21% (107) had diploma-level degrees, 12% (59) had postgraduate degrees, and 7% (38) had only a primary education.

### 4.2. Goodness of Measures

To test whether the items used in the survey to measure the QoL, measured the construct, an exploratory factor analysis (varimax rotation method) was conducted, and the results are presented in Table 1. The factor analysis results in the establishment of the latent factor of QoL confirmed the validity and reliability of the latent factor, by evaluating the convergent validity (factor loadings of respective measured items > 0.50), the discriminate validity (average variance extracted > square of the correlation value), and Cronbach’s alpha coefficients (lowest acceptable limit of coefficient ranges between 0.6 and 0.7) [55]. High reliability (internal consistency) of a latent factor confirms that the outcome of a study is accurate.

### 4.3. Descriptive Statistics

The nature and degree of six aspects of QoL alongside the total scale are presented in Table 1. The respondents were asked to rate their current levels to reflect their feelings and thoughts for each item on the scale during “the year of COVID-19”. The findings revealed that COVID-19 generally impacted the QoL of Saudi households to a significant extent (Mean = 4.25, SD = 0.592). The largest impact of COVID-19 was on the affection domain (feelings, emotions, etc.) with Mean = 4.29 (SD = 0.876) and smallest on life status with the Mean = 4.16 (SD = 0.882). Overall, this result provides acceptable evidence that COVID-19 has negatively impacted QoL, which supports Hypothesis 1 (H1).

A one-way ANOVA was conducted to determine the variation of the impact of COVID-19 according to region, area of residence, the education level of the head of household, the gender of the head of the household, the age of the head of household, the income of the head of household, the occupation type/sector of the head of household, family size, and the number of working family members. The results are presented in Table 2, Table 3, Table 4 and Table 5.

The ANOVA revealed that the detrimental effects of COVID-19 on each aspect of and overall QoL exhibited statistically significant differences by region. We can see in Table 2 that the significance values are less than 0.01 (i.e., *p* < 0.01) and, therefore, there is a statistically significant difference in the mean values of regions. In Tukey’s HSD (Honestly significant difference) test for multiple comparisons, the mean values for the deterioration of each aspect of QoL as well as overall QoL were significantly different between the central and other regions (except the western region) as well as between western and other regions (except the central region) (Table 2). The result indicated that households in the central and western regions recorded significantly higher (*p* ≤ 0.01) levels of deterioration of QoL compared with other regions. A potential reason for this result could be the levels of disease intensity, as this affects QoL; the death toll and other impacts of COVID-19 have varied significantly across regions. Since different regions have experienced varying levels of disease intensity, the impact of COVID-19 on QoL varied accordingly. The ANOVA of survey responses (Table 2) also indicated significant variation in the impact of COVID-19 on QoL across regions, driven by differences in demographic characteristics.

A second potential reason for this result could be the level of anti-COVID-19 measures and related efforts taken by authorities. The regression analysis of QoL included all five regions to observe how QoL has varied. Our empirical results suggest that anti-COVID-19 measures and efforts in all five regions negatively affected QoL. However, they affected it significantly in two regions, namely the central region, e.g., Riyadh and Qassim (X9) and the western region, for example, Makkah and Madinah (X10). This could be due to the fact that these two regions attract the most religious travelers (both local and international) for many purposes, mainly for Islamic pilgrimages. Therefore, massive anti-COVID 19 measures and efforts were taken to protect locals and travelers, which impacted livelihoods and opportunities and removed household earnings in these regions, resulting in significant negative impacts on QoL.

ANOVA also uncovered that, in terms of the decline in each dimension of QoL as well as overall QoL, there was a statistically significant (*p* < 0.01) variation of among residential areas (Table 2). Similarly, ANOVA revealed significant differences in the decline in QoL due to COVID-19 by head of family age. In Tukey’s HSD test for multiple comparisons, the mean values of the deterioration of each aspect of QoL, as well as overall QoL, were significantly different between household heads aged ≥55 years and household heads aged from 35 to 54 years (*p* < 0.01) and under 35 years (*p* < 0.01), except for depression. However, no significant difference was found between household heads aged from 35 to 54 years and those aged less than 35 years for most aspects of QoL (Table 2). The results indicated that households in urban areas and headed by older people recorded significantly higher (*p* ≤ 0.01) levels of deterioration of QoL compared with other households. The impact of COVID-19 concerning the individually tested domain (individual items of QoL) was also significantly higher (*p* ≤ 0.01) on households with these features compared with other households.

ANOVA also revealed variation in QoL decline for each dimension as well as for overall QoL across gender, education level, and income level for the heads of households. Statistically significant differences were present. We can see in Table 3 that the significance values are less than 0.05 (i.e., *p* < 0.05). There were statistically significant differences in the mean values for men vs. women (*p* < 0.05), education (*p* < 0.05), and income (*p* < 0.01) of heads of households as a whole except for depression. Tukey’s HSD test for multiple comparisons revealed that male-led households with primary and secondary educational qualifications reported a significantly (*p* ≤ 0.01) greater decline in QoL than others (Table 3). Furthermore, the results showed that the QoL of lower- and middle-income households was significantly worse than that of higher-income households. However, the impact of COVID-19 concerning mental health and depression did not differ significantly according to the education level of the head of the household.

Additional ANOVA was conducted to test the variation in the reported decline in each dimension of QoL as well as overall QoL by occupation sector and family size. There were no statistically significant differences (*p* > 0.05) in QoL decline due to COVID-19 according to occupation sectors, while statistically significant differences (*p* < 0.05) were found in terms of the number of family members living together, except in the physical health and tense aspects (Table 4). Tukey’s HSD test for multiple comparisons showed that regarding QoL, large-size households reported significantly (*p* ≤ 0.01) higher levels of decline than others, but no significant difference was found among households according to the occupation sector of the household head. The impact of COVID-19 concerning the individual QoL items was also significantly higher (*p* ≤ 0.05) for households with the same features compared with other households, except in physical health and tense domains.

The additional ANOVA tests were conducted to measure variation in the decline for each dimension of QoL as well as overall QoL across occupation type and the number of working family members. There were statistically significant differences (*p* > 0.05) in QoL decline for both groups. Tukey’s HSD test for multiple comparisons revealed that during the pandemic, the individual aspects of QoL as well as overall QoL of those households headed by an unemployed or self-employed individual was significantly lower (*p* ≤ 0.01) than that of others (Table 5). Table 5 indicates that the QoL of households with one working member declined at a significantly (*p* ≤ 0.01) higher rate compared with households that had more than one working member. Overall, most households felt helpless regardless of socio-demographic variables. Thus, the results of ANOVA provide evidence that the socio-demographic characteristics of the participants were significantly associated with the overall QoL score and individual QoL items, with the exception of the occupation sector of the head of household, which supported H2.

To further analyze H2, multiple regression analyses were conducted using the total QoL score (created by averaging scores for all items). The R^2^ statistic for the regression showed that more than 40% of the variance in QoL was predictable for all the predicted variables. The results showed a good fit for these data. The F-test showed a significant result (*p* < 0.01), which implied that all the variables in the model were significant predictors of QoL. Table 6 shows the results of the regression analyses, which uncovered significant variation in the effects of the coronavirus pandemic on household QoL across demographic factors, such as age, gender, employment status, region, area of residence, income level, number of working family members, and family size.

The variable on the gender status of the household head (X_1_) proved to bear a positive sign with a statistical significance at the 0.05 level. This means that the QoL of male-headed households deteriorated more than that of female-headed households due to the economic crisis caused by the pandemic. The regression coefficient 0.101 measures that holding other variables constant, a positive change can be seen in the value QoL by per unit positive change of households headed by males during the crisis. Similarly, the residential area variable (X_2_) as a determinant also proved to be statistically significant at the *p* ≤ 0.01 level and positively related to QoL. This finding indicates that households in urban areas were more seriously affected than others, when other variables were treated as control variables. The regression coefficient of this variable, 0.169, means that by holding other variables constant, QoL decreased by an average of 0.169% for one unit increase of the household that was living in urban areas.

Similarly, the variables of household head education level categories X_18_ (up to primary education), X_19_ (secondary), X_20_ (bachelor), and X_21_ (diploma) proved to have no significant difference in QoL deterioration across the education levels of household heads, while the living area variables X_9_ and X_10_ proved that when other factors were held constant, QoL declined in households in the central (Riyadh and Qassim) and western (Makkah and Madinah) regions significantly (at *p* ≤ 0.01), exceeding that of the households in other regions. Moreover, the decline in QoL in unemployed- and self-employed-headed households was significantly higher than in others (at *p* ≤ 0.01 and *p* ≤ 0.05, respectively). The regression results proved that the QoL of the households headed by informal sector employees was worsened significantly more (*p* ≤ 0.05) than that of others, while the QoL of households headed by construction employees was less damaged.

Finally, the results indicated that the diminished QoL of households with aged heads, large family sizes, small numbers of working family members, recently unemployed heads, and lost ability to repay debts was significantly more severe than that of others. Overall, Table 6 shows that demographic variables were significantly related to the deterioration of QoL caused by COVID-19, which strongly supports the second hypothesis (H2) of this study.

Six ordered Probit models were run discretely to further investigate which demographic characteristics most significantly affected individual aspects of QoL. A summary drawn from the models is presented in Table 7, where individual model strength measured by the Nagelkerke R^2^ value and the most significant demographic characteristics affecting individual aspects of QoL are documented. In terms of the model fitness indicator, however, the health aspect of QoL was the most concerning aspect of the six, while the depression aspect was the least concerning. Furthermore, no demographic characteristic was found to be consistent and significant across all six aspects of QoL, implying that no single demographic characteristic was a dominant factor in QoL. Evidently, occupation status, number of family members, age of household head, and residential region (province) were the most important factors affecting QoL across all regions.

## 5. Discussion

It is universally recognized that the effects of COVID-19 are significantly influencing human lives. Therefore, it is important to examine how different segments of society are affected and to establish a concrete, comprehensive social safety net. To the best of our knowledge, our study is the first to examine the immediate impacts of COVID-19 on the QoL of households in Saudi Arabia. Since the pandemic is not yet over (and the number of active cases fluctuates), the pandemic may continue to cause fear and anxiety among households in Saudi Arabia. However, the relevant authorities were made aware of the severity of the virus and took prompt action to protect the residents of the country.

This study found that the mean score on the COV19-QoL scale was 4.25, indicating that Saudi households were generally severely affected. Most households reported that their QoL was worse compared to the pre-COVID-19 period. Therefore, the first hypothesis (H1), that COVID-19 adversely affects the QoL of Saudi households, is accepted. This finding supports the findings of earlier studies [9,27,30,39,40,43,57,58,59,60]. This study also indicates that the sample of households exhibited a COVID-19 impact on QoL, as a whole, greater than what has been reported in the aforementioned studies, which found moderate impacts on well-being and living standards. There could be several potential reasons for this finding. Firstly, due to cultural differences, levels of health may differ by country. Secondly, age, gender structure, and health status also vary by country. Thirdly, the study period could have an impact as earlier studies were conducted at the early stage of pandemic when the outbreak was not regarded as severe, and lockdowns were not applied at the early stage. However, we collected our data about one and half years later, when the coronavirus had spread worldwide, with millions of people dead or struggling in healthcare centers and with lockdowns applied around the world, including in Saudi Arabia. Fourthly, social life in Saudi Arabia is imperative, and when isolation policies were imposed by the government, society suffered psychologically and mentally. Fifthly, the important services were stopped in Saudi Arabia, e.g., entertainment (cinemas) and travel (flights). Sixthly, hospitals were filled with confirmed cases, which made receiving medical care difficult. Seventhly, health clubs (gyms) were closed, and other sports centers in Saudi Arabia were not ready to do sports, thereby decreasing the strength of immunity systems. These factors might have aggravated the impact of COVID-19.

Notably, regarding regional and area dimensions, households in some regions were more affected by COVID-19 than those in others. For example, households in the western and central regions and in urban areas reported a significantly greater impact of COVID-19 on QoL compared to others. Therefore, this element of the second hypothesis (H2), that the impact of the pandemic on the QoL of households varied by region, is accepted. QoL clearly varied geographically with variations in relative QoL occurring for any given place; there was a significant connection between the magnitude of the shock and regions that were relatively well off, with booming modern economic sectors before the COVID-19 crisis. Based on the results of the statistical analysis, we can say that households in the western and central regions experienced greater deterioration of QoL compared to other regions because these regions were relatively prosperous. Another potential reason could be their economic dependency on tourism. Religious tourism is central to their economy and the main engine for generating income, as Hajj and Umrah pilgrimages play key roles in these two regions. This sector was terribly affected by the outbreak due to several lockdowns and various restrictions on the Umrah and Hajj by the Saudi government and other countries. Restrictions stopped people from traveling to these two holy places, which impacted livelihoods and opportunities and removed household earnings in these two regions. Households facing acute economic crises may be less willing to follow social distancing rules and instead seek out income-generating opportunities, even in crowded and epidemiologically risky places. Moreover, the western and central regions have the largest populations of these regions. In addition, they have more facilities, such as malls, shops, restaurants, and health clubs, which increases the risk of increasing the number of COVID-19 cases. This finding supports the findings of Liew [61], Foo et al. [62], Wu et al. [63], Sharma and Nicolau [64], and Polyzos et al. [65].

The findings of this study showed that the QoL of households in urban areas was more seriously affected by COVID-19. Therefore, this element of the second hypothesis (H2) (that the impact of the COVID-19 outbreaks on the QoL of households significantly differed according to residential area) was accepted. There could be several reasons for this outcome. The first is greater exposure to risks and economic difficulties due to high density and the preexisting conditions of urban households. The population of the KSA is not uniformly allocated and is clustered in particular regions and urban communities, and almost 82% of the population lives in urban areas [13]. In urban areas, communities that produce services (which are typically non-tradable) have seen their income decline more than in other areas, as the economic crisis caused by COVID-19 has mainly hit the formal economy. The second reason could be that most of the respondents with urban households from vulnerable and marginalized groups were those expected to be severely affected by COVID-19, such as the urban poor who live in densely populated informal urban areas with poor living and sanitary conditions and with limited access to services. In contrast, rural households, especially those living in remote areas and agricultural communities, were most protected from the harmful effects of the economic crisis caused by the spread of COVID-19 due to minimal spread. Hence, rural households were psychologically less affected by the pandemic. These communities are also likely to have less interaction with monetary sectors of the economy. This result supports the findings of Wade [66] and Napierała et al. [67].

The QoL in female-headed households was generally less affected by the crisis. Interestingly, this finding completely contrasts with the findings of previous studies [35,68,69] in which female-headed households were worse affected by the pandemic crisis. A potential reason for this finding could be the government’s ongoing attempt to achieve the goals of Saudi Arabia’s Vision 2030. One of the main objectives of Vision 2030 is women’s empowerment through providing jobs in all sectors in Saudi Arabia, especially in the public sector, which was least affected by the pandemic. In addition, Saudi Arabia has launched non-contributory unemployment benefit programs whose recipients are mainly women [70]. These attempts have elevated the financial status of Saudi women, while male-headed households have experienced significant reductions in employment and increases in burdens. Our findings revealed that households whose heads were older, that had fewer income earners, and that had more dependents reported a significantly greater impact of COVID-19 on QoL compared to other households. This means that, holding other factors constant, QoL has been affected in larger households more so than in smaller households. This result parallels general economic theory, which maintains that there is a negative relationship between family size and QoL. The empirical evidence in our study also showed that family identity (such as the age of the household head, family size, income level, and number of earning family members) is a significant predictor of QoL. This result supports the assertions of the UN of KSA [13], Stevenson et al. [71], and Kharshiing et al. [38]. Briefly, they have stated that family identification and structure have been seen to significantly impact QoL during the economic crisis caused by COVID-19. Perhaps it can be assumed that households with aged heads, large family sizes, small numbers of working family members, recently unemployed heads, and lost ability to repay debts were unable to cope better than other households. This finding supports the findings of other studies [30,34,38]. Thus, the findings in this study validate Hypothesis 2 (H2).

## 6. Conclusions, Limitations, and Recommendations

This empirical study delivers insight into the initial micro-level effects of COVID-19 in Saudi Arabia. Using primary data, it uncovered how COVID-19 affected the QoL of households. It showed that COVID-19 severely affected the QoL of households regardless of socio-demographic features. According to the research respondents, COVID-19 had the most detrimental effect on the domain of affection (such as feelings and emotions), and the second-most affected domain was physical health and social status of life. It also found evidence that COVID-19 has had a varied effect according to demographic characteristics, such as regional dimensions, area dimensions, age, household size, number of income earners, gender, and employment status. The study confirmed that male-headed households were more greatly affected regarding QoL compared to their female-headed counterparts, urban households were more seriously affected than rural households, western and central region households were more negatively affected than other regions, and the adverse effects were much stronger for unemployed and self-employed households relative to others.

The evidence presented here is on the immediate impact of the crisis caused by COVID-19. The medium- and long-term impacts of the pandemic cannot be known. However, its effects on mental and physical health may be felt for many years to come. Though the Saudi government has initiated several immediate emergency and recovery actions, there is still room to improve the social safety net. Therefore, we are providing recommendations regarding the needs of both today and tomorrow to prevent future pandemics and remedy inequalities, primarily through policy recommendations for a solid, comprehensive social safety net directly and indirectly linked to sustainable economic development. Firstly, there is an urgent need for the country to broaden its social protection programs by increasing government assistance, or at least continuing the existing assistance, to both the newly and existing underprivileged. Secondly, solutions and policies should be modified or designed to address issues from a systemic perspective so as to provide the most comprehensive and prudent solutions to difficulties induced by the current pandemic to help people live their lives effectively, despite COVID-19. Thirdly, a robust mechanism that ensures that capital disbursement is aimed at the most vulnerable groups and businesses needs to be developed and enforced. The fourth suggestion is to allocate consultants for employees in organizations (e.g., companies and corporations) to provide advice on handling COVID-19. Finally, the following measures are imperative: Encouraging households to find multiple sources of income and encouraging steps to stop the spread of the virus by following preventive steps (e.g., social distancing, wearing a mask, using hand sanitizer, hand-washing) to reduce the number of cases and thus avoid the negative effects of the virus on QoL.

The present study has a few strengths. To our knowledge, it is the first to offer a unique opportunity to uncover the effects of COVID-19 at the micro-level. Furthermore, this study was conducted at the household level when the virus had spread fatally throughout Saudi Arabia, and lockdowns had been applied throughout the country. This is vital, as the study gathered initial data about the impacts of COVID-19 on QoL. The study might therefore help policymakers understand what is needed to concurrently address immediate, medium-term, and long-term needs. In addition, the study contributes to the literature on the impact of COVID-19 and could serve as a starting point or reference for future studies. Finally, the discoveries emphasize the significance of generating on-the-ground survey data to track the well-being of households during the crisis, to accumulate the information required to construct evidence-based policy responses.

However, this study has limitations. The first is the electronic data collection method (through survey), which limits availability to a greater population. Second, most of the respondents were male and middle-income, thus limiting the generalizability of the findings. Finally, due to the complex nature of the ongoing viral pandemic, the study’s one-time cross-sectional data may not be sufficient for clarifying QoL. A randomized prospective study could better determine correlation and causation. Moreover, due to the large size of Saudi Arabia, a large sample from different areas is needed to verify the results. Therefore, we suggest that future research on the linkages of QoL and COVID-19 shocks, considering all other components of QoL that address a holistic relationship between QoL and pandemic shocks, would contribute toward a greater understanding of their initial impact.

## Figures and Tables

**Table 1:** Results for Principal Components Analysis, reliability, and internal consistency, and descriptive statistical values of the COV19-QoL scale.

Items and Scale	Loadings	Mean	SD	Cronbach’s Alpha	Average (AVE)	(r^2^)^2^
Quality of life scale (QoL)		**4.25**	**0.592**	**0.744**	**0.439**	**0.153**
My mental health has deteriorated.	0.701	4.25	0.957			
I feel more depressed than before.	0.698	4.29	0.876			
I feel more tense than before.	0.690	4.27	0.886			
My physical health has deteriorated.	0.660	4.26	0.887			
I feel that my personal safety is at risk.	0.616	4.27	0.879			
My condition of life is lower than before.	0.604	4.16	0.882			

**Table 2:** ANOVA for mean scores of respondents on QoL items and QoL scale classified by living regions, living areas, and age of household head.

Items and Scale	Living Region ^a^							Sig. Diff. Groups	Living Area ^b^				Sig. Diff. Groups	Age of Household Head ^c^			Sig. Diff. Groups		
	1 (*n* = 106)	2 (*n* = 111)	3 (*n* = 103)	4 (*n* = 91)	5 (*n* = 95)	F Value	*p* Value		1 (*n* = 283)	2 (*n* = 223)	F Value	*p* Value		1 (*n* = 111)	2 (*n* = 246)	3 (*n* = 149)		F Value	*p* Value
The condition of my life is worse than before.	4.36	4.42	3.98	3.88	4.12	7.62	0.000	(1–3) ** (1–4) ** (2–3) ** (2–4) **	4.33	3.95	24.38	0.000	(1–2) **	3.94	4.11	4.42	(1–3) ** (2–3) **	10.96	0.000
My mental health has deteriorated.	4.57	4.55	3.98	4.09	4.02	10.39	0.000	(1–3) ** (1–4) ** (1–5) ** (2–3) ** (2–4) ** (2–5) **	4.42	4.04	20.85	0.000	(1–2) **	4.09	4.13	4.59	(1–3) ** (2–3) **	13.71	0.000
My physical health has deteriorated.	4.51	4.48	4.01	4.08	4.18	7.33	0.000	(1–3) ** (1–4) ** (2–3) ** (2–4) **	4.40	4.09	16.10	0.000	(1–2) **	3.96	4.25	4.50	(1–2) ** (1–3) ** (2–3) *	11.99	0.000
I feel more tense than before.	4.50	4.49	4.07	4.15	4.12	6.12	0.000	(1–3) ** (1–4) * (1–5) * (2–3) ** (2–4) * (2–5) *	4.41	4.10	15.36	0.000	(1–2) **	3.96	4.20	4.63	(1–2) * (1–3) ** (2–3) **	21.33	0.000
I feel more depressed than before.	4.51	4.52	4.06	4.12	4.19	6.87	0.000	(1–3) ** (1–4) ** (2–3) ** (2–4) ** (2–5) *	4.43	4.11	17.93	0.000	(1–2) **	4.16	4.25	4.45	(1–3) *	3.93	0.020
I feel that my personal safety is at risk.	4.45	4.45	4.05	4.19	4.20	4.41	0.002	(1–3) ** (2–3) **	4.40	4.12	13.19	0.000	(1–2) **	4.05	4.23	4.52	(1–3) ** (2–3) **	10.47	0.000
Quality of life (QoL Scale)	4.48	4.49	4.02	4.08	4.14	16.66	0.000	(1–3) ** (1–4) ** (1–5) ** (2–3) ** (2–4) ** (2–5) **	4.40	4.07	42.50	0.000	(1–2) **	4.03	4.19	4.52	(1–2) * (1–3) ** (2–3) **	26.74	0.000

Note: ^a^ 1—Central Region (Riyadh and Qassim), 2—West Region (Makkah and Madinah), 3—Eastern Region (Eastern Province), 4—Southern Region (Asir, Jazan, Najran and Baha), 5—Northern Region (Northern Boarders, Hail and Jouf); ^b^ 1—Urban, 2—Rural; ^c^ 1—less than 35 years, 2—35 to 54 years, 3—55 and more years; ** *p* ≤ 0.01 level, * *p* ≤ 0.05 level.

**Table 3:** ANOVA for mean scores of respondents on QoL items and QoL scale classified by education levels, home ownership, and gender.

Items and Scale	Level of Education of Household Head ^a^					F Value	*p* Value	Sig. Diff. Groups	Gender of Household Head ^c^		F Value	*p* Value	Sig. Diff. Groups	Income Levels of Household Head ^b^			F Value	*p* Value	Sig. Diff. Groups
	1 (*n* = 38)	2 (*n* = 110)	3 (*n* = 107)	4 (*n* = 192)	5 (*n* = 59)				1 (*n* = 146)	2 (*n* = 360)				1 (*n* = 180)	2 (*n* = 294)	3 (*n* = 32)			
The condition of my life is worse than before.	4.55	4.40	4.08	4.04	4.02	5.55	0.000	(1–3) * (1–4) ** (1–5) * (2–4) ** (2–5) *	3.98	8.48	0.000	0.003	(1–2) **	4.37	4.06	3.91	8.48	0.000	(1–2) ** (1–3) **
My mental health has deteriorated.	4.58	4.45	4.18	4.18	4.08	3.20	0.013	None	3.98	5.28	0.005	0.000	(1–2) **	4.38	4.22	3.81	5.28	0.005	(1–3) ** (2–3) **
My physical health has deteriorated.	4.39	4.45	4.23	4.23	3.98	2.99	0.019	(2–5) **	4.12	6.41	0.002	0.020	(1–2) *	4.41	4.22	3.84	6.41	0.002	(1–3) **
I feel more tense than before.	4.53	4.48	4.22	4.17	4.15	3.34	0.010	(2–4) *	4.07	9.82	0.000	0.001	(1–2) **	4.51	4.15	4.13	9.82	0.000	(1–2) **
I feel more depressed than before.	4.34	4.45	4.27	4.23	4.19	1.46	0.214	None	4.01	7.74	0.000	0.000	(1–2) **	4.48	4.20	4.00	7.74	0.000	(1–2) ** (1–3) **
I feel that my personal safety is at risk.	4.37	4.47	4.24	4.23	4.05	2.65	0.032	(2–5) *	4.13	7.86	0.000	0.018	(1–2) *	4.45	4.21	3.88	7.86	0.000	(1–2) ** (1–3) **
Quality of life (QoL Scale)	4.46	4.45	4.21	4.18	4.08	6.66	0.000	(1–4) * (1–5) ** (2–3) * (2–4) ** (2–5) **	4.05	16.43	0.000	0.000	(1–2) **	4.43	4.17	3.93	16.43	0.000	(1–2) ** (1–3) ** (2–3) *

Note: ^a^ 1—Up to primary, 2—Secondary 3—Diploma, 4—Bachelor, 5—Master’s/PhD; ^b^ 1—less than 3800 SR, 2—3800 to 38,200 SR, 3—More than 38,200SR; ^c^ 1—Female, 2—Male; ** *p* ≤ 0.01 level, * *p* ≤ 0.05 level.

**Table 4:** ANOVA for mean scores of respondents on QoL items and QoL scale classified by number of family members living together and occupation sector.

Items and Scale	Occupation Sector ^b^						F Value	*p* Value	Sig. Diff. Groups	Number of Family Members Living Together ^a^			F Value	*p* Value	Sig. Diff. Groups
	1 (*n* = 61)	2 (*n* = 133)	3 (*n* = 42)	4 (*n* = 150)	5 (*n* = 48)	6 (*n* = 72)				1 (*n* = 169)	2 (*n* = 247)	3 (*n* = 90)			
The condition of my life is worse than before.	4.43	4.14	4.00	4.13	4.15	4.15	1.43	0.211	None	4.04	4.15	4.42	5.60	0.004	(1–3) ** (2–3) *
My mental health has deteriorated.	4.31	4.15	4.36	4.32	4.04	4.35	1.21	0.304	None	4.18	4.21	4.51	4.01	0.019	(1–3) * (2–3) *
My physical health has deteriorated.	4.30	4.29	4.21	4.25	4.27	4.31	0.70	0.625	None	4.24	4.23	4.48	1.61	0.201	None
I feel more tense than before.	4.26	4.30	4.38	4.27	4.06	4.24	0.09	0.994	None	4.28	4.20	4.39	2.90	0.056	None
I feel more depressed than before.	4.30	4.24	4.19	4.36	4.25	4.32	0.42	0.836	None	4.22	4.21	4.66	9.83	0.000	(1–3) ** (2–3) **
I feel that my personal safety is at risk.	4.31	4.27	4.33	4.23	4.27	4.31	0.14	0.982	None	4.26	4.17	4.59	7.71	0.001	(1–3) ** (2–3) **
Quality of life (QoL Scale)	4.32	4.23	4.25	4.26	4.17	4.28	0.37	0.867	None	4.20	4.19	4.51	10.45	0.000	(1–3) ** (2–3) **

Note: ^a^ 1—Less than 5 members, 2—5 to 10 members, 3—More than 10 members; ^b^ 1—Agriculture, 2—Informal, 3—Construction, 4—Services, 5—Manufacturing, 6—Business; ** *p* ≤ 0.01 level, * *p* ≤ 0.05 level.

**Table 5:** ANOVA for mean scores of respondents on QoL items and QoL scale classified by number of working family members and types of occupation.

Items and Scale	Types of Occupation ^b^					F Value	*p* Value	Sig. Diff. Groups	Number of Working Family Members ^a^			F Value	*p* Value	Sig. Diff. Groups
	1 (*n* = 66)	2 (*n* = 149)	3 (*n* = 168)	4 (*n* = 81)	5 (*n* = 42)				1 (*n* = 169)	2 (*n* = 247)	3 (*n* = 90)			
The condition of my life is worse than before.	4.33	4.38	4.07	4.01	3.81	5.93	0.000	(1–5) * (2–3) ** (2–4) * (2–5) **	4.34	4.21	3.82	12.45	0.000	(1–3) ** (2–3) **
My mental health has deteriorated.	4.39	4.48	4.15	3.94	4.24	5.33	0.000	(1–4) * (2–3) * (2–4) **	4.57	4.20	3.96	14.85	0.000	(1–2) ** (1–3) **
My physical health has deteriorated.	4.26	4.42	4.23	4.05	4.21	2.49	0.042	(2–4) *	4.48	4.22	4.05	8.52	0.000	(1–2) ** (1–3) **
I feel more tense than before.	4.48	4.43	4.16	4.10	4.19	3.73	0.005	(2–3) * (2–4) *	4.54	4.26	3.95	15.03	0.000	(1–2) ** (1–3) ** (2–3) **
I feel more depressed than before.	4.38	4.49	4.24	4.09	4.05	4.25	0.002	(2–4) ** (2–5) *	4.56	4.27	3.98	14.65	0.000	(1–2) ** (1–3) ** (2–3) **
I feel that my personal safety is at risk.	4.36	4.48	4.17	4.06	4.21	4.17	0.003	(2–3) ** (2–4) **	4.52	4.25	4.00	11.69	0.000	(1–2) ** (1–3) ** (2–3) *
Quality of life (QoL Scale)	4.37	4.45	4.17	4.04	4.12	9.17	0.000	(1–4) ** (2–3) ** (2–4) ** (2–5) **	4.50	4.23	3.96	3023	0.000	(1–2) ** (1–3) ** (2–3) **

Note: ^a^ 1—Working 1 member, 2—Working 2 members, 3—Working more than 2 members; ^b^ 1—Unemployed, 2—Self-employed, 3—Employee, 4—Employer, 5—Retired; ** *p* ≤ 0.01 level, * *p* ≤ 0.05 level.

**Table 6:** Results of the multiple regression analysis for QoL.

**Variable**	**Estimated Coefficient (B)**	**Std. Err.**
Constant	3.336 (22.429) **	0.149
Gender of households (X_1_) (1 for male and 0 for female)	0.101 (2.019) *	0.050
Residential area (X_2_) (1 for Urban and 0 for Rural)	0.167 (3.678) **	0.045
Ownership of house (X_3_) (1 for owned and 0 rented)	0.003 (0.078)NS	0.044
Terminated or lost jobs or decreased wages since COVID-19 crisis started (X_4_) (1 for yes and 0 for no)	0.114 (1.992) *	0.060
Family expenditures affected unfavorably since COVID-19 started (X_5_) (1 for yes and 0 for no)	0.137 (2.906) **	0.047
The repayment of the loan been affected unfavorably due to economic crisis that occurred because of COVID-19 (X_6_) (1 for yes and 0 for no)	0.114 (2.062) *	0.055
**Age of Households’ Head**		
From 35 to 54 years (X_7_)	0.124 (2.262) *	0.055
55 and more years (X_8_)	0.237 (3.599) **	0.066
**Households’ Living Region**		
Central Region (Riyadh and Qassim) (X_9_)	0.348 (5.174) **	0.067
West Region (Makkah and Madinah) (X_10_)	0.356 (5.081) **	0.070
Eastern Region (Eastern Province) (X_11_)	0.084 (1.219)NS	0.069
Northern Region (Northern Boarders, Hail and Jouf) (X_13_)	0.131 (1.866)NS	0.070
**Household Heads’ Primary Occupation**		
Unemployed (X_14_)	0.215 (2.601) **	0.083
Self-employed (X_15_)	0.148 (2.078) *	0.071
Employee (X_16_)	0.089 (1.347)NS	0.066
Retired (X_17_)	0.045 (0.492)NS	0.092
**Household Heads’ Education Level**		
Up to primary (X_18_)	0.089 (0.904)NS	0.102
Secondary (X_19_)	0.050 (0.765)NS	0.076
Diploma (X_20_)	−0.009 (−0.279)NS	0.072
Bachelor (X_21_)	0.029 (0.029)NS	0.079
**Number of Family Members**		
Less than 5 members (X_22_)	−0.154 (−2.525) **	0.065
5 to 10 members (X_23_)	−0.163 (−2.685) **	0.061
**Number of Working Family Members**		
Working family member 1 (X_24_)	0.298 (4.626) **	0.064
Working family member 2 (X_25_)	0.133 (2.308) *	0.058
**Occupation Sector of Households’ Head**		
Agriculture (X_26_)	0.082 (0.931)NS	0.088
Informal (X_27_)	0.143 (2.438)*	0.059
Construction (X_28_)	−0.142 (−1.664)NS	0.085
Service (X_29_)	0.070 (1.170)NS	0.060
Business (X_30_)	0.048 (0.780)NS	0.062
**Number of Observation**	506	
**d.f**	31	
**R^2^**	0.411	
**Adjusted R^2^**	0.373	
**F**	10.687 **	

Note: Figures in parentheses denote the t-values of the regression coefficients; ** indicate significance at *p* ≤ 0.01 level; * indicate significance at *p* ≤ 0.05 level. Reference dummy variables: Head of households with less than 35 years of age, Southern region, employer, Master’s/PhD education, more than 10 family members, working family members of more than 2, and manufacturing sector as a reference variable.

**Table 7:** Summary of ordered Probit models on the aspects of quality of life that are driven by demographic characteristics of respondents.

Aspect of QoL Used as Dependent Variable in Ordered Probit Model	Nagelkerke R^2^	Demographic Characteristics That Most Significantly Affect the Aspect of QoL
My life condition is worse than before.	0.308	1. Residential Region 2. No. of family members working 3. Monthly income
My mental health has deteriorated.	0.334	1. Residential Region 2. Occupation status
My physical health has deteriorated.	0.259	1. Age of household head
I feel more tense than before.	0.288	1. Age of household head
I feel more depressed than before.	0.267	1. No. of family members
I feel that my personal safety is at risk.	0.269	1. No. of family members 2. Occupation status

## Data Availability

A data set that is relevant to this article is available to the editorial office any time it is required.
